# Efficacy and safety of safinamide in Parkinson’s disease patients with motor fluctuations without levodopa dosage escalation over 18 weeks: KEEP study

**DOI:** 10.1007/s00702-024-02851-6

**Published:** 2024-11-14

**Authors:** Eungseok Oh, Sang-Myeong Cheon, Jin Whan Cho, Young Hee Sung, Joong-Seok Kim, Hae-Won Shin, Jong-Min Kim, Mee Young Park, Do-Young Kwon, Hyeo Ma, Jeong-Ho Park, Seong-Beom Koh, Seong-Min Choi, Jinse Park, Phil Hyu Lee, Tae-Beom Ahn, Sang Jin Kim, Chul Hyoung Lyoo, Ho-Won Lee, Jieun Kim, Yoona Lee, Jong Sam Baik

**Affiliations:** 1 Department of Neurology, Chungnam National University College of Medicine and Hospital, Daejeon, Republic of Korea; 2 Department of Neurology, School of Medicine, Dong-A University, Busan, Republic of Korea; 3 Department of Neurology, Samsung Medical Center, Sungkyunkwan University School of Medicine, Seoul, Republic of Korea; 4 Department of Neurology, Gil Medical Center, Gachon University College of Medicine, Incheon, Republic of Korea; 5 Department of Neurology, College of Medicine, The Catholic University of Korea, Seoul St. Mary’s Hospital, Seoul, Republic of Korea; 6 Department of Neurology, Chung-Ang University Hospital, Seoul, Republic of Korea; 7 Departments of Neurology, Seoul National University Bundang Hospital, Seoul National University College of Medicine, Seongnam, Republic of Korea; 8 Department of Neurology, Yeungnam University College of Medicine, Daegu, Republic of Korea; 9 Department of Neurology College of Medicine, Korea University Ansan Hospital, Ansan, Republic of Korea; 10 Department of Neurology, Hallym University Sacred Heart Hospital, Hallym University College of Medicine, Anyang, Republic of Korea; 11 Department of Neurology, College of Medicine, Soonchunhyang University Bucheon Hospital, Gyeonggi-do, Republic of Korea; 12 Department of Neurology, Korea University Guro Hospital, Korea University College of Medicine, Seoul, Republic of Korea; 13 Department of Neurology, Chonnam National University Hospital, Chonnam National University Medical School, Gwangju, Republic of Korea; 14 Department of Neurology, Inje University Haeundae Paik Hospital, Busan, Republic of Korea; 15 Department of Neurology, Yonsei University College of Medicine, Seoul, Republic of Korea; 16 Department of Neurology, Kyung Hee University Hospital, Kyung Hee University College of Medicine, Seoul, Republic of Korea; 17 Department of Neurology, Busan Paik Hospital, Inje University College of Medicine, Busan, Republic of Korea; 18 Department of Neurology, Gangnam Severance Hospital, Yonsei University College of Medicine, Seoul, Republic of Korea; 19 Department of Neurology, School of Medicine, Kyungpook National University, Daegu, Republic of Korea; 20 Department of Medical, Eisai Korea Inc., Seoul, Republic of Korea; 21 Department of Neurology, Inje University Sanggye Paik Hospital, Seoul, Republic of Korea

**Keywords:** Safinamide, Motor fluctuation, PDQ-39, QoL, Pain, Korea

## Abstract

This multicentre, prospective, single-arm study evaluated safinamide as add-on therapy to levodopa in Korean patients with Parkinson’s disease (PD) with motor fluctuations with ≥ 1.5 h of “off” time daily, who took levodopa ≥ 3 times/day (*n* = 199). Baseline levodopa and dopamine agonist doses were maintained without escalation during the 18-week treatment period. Participants received safinamide 50 mg/day for 2 weeks and 100 mg/day thereafter. PD diaries and questionnaires (Parkinson’s Disease Questionnaire, PDQ-39; Movement Disorder Society-Sponsored Revision of the Unified Parkinson’s Disease Rating Scale, MDS–UPDRS part 3 and part 4; King’s Parkinson’s Disease Pain Scale, KPPS; Mini-Mental State Examination, MMSE) were assessed at baseline and at week 18. Treatment-emergent adverse events (TEAEs) were recorded. Mean disease duration was 6.6 years, and mean levodopa equivalent daily dose was 721.1 mg/day. At week 18, significant improvements from baseline were seen for the co-primary endpoints, mean daily “off” time (− 1.3 ± 2.4 h, *p *< 0.001) and quality of life (QoL) based on PDQ-39 summary index (− 2.7 ± 10.3, *p *< 0.001), Moreover, significant improvements were seen in motor symptoms and motor complications (MDS-UPDRS part 3 and 4), daily “on” time without dyskinesia (all *p *< 0.001) and pain (KPPS; *p* = 0.013). TEAEs occurred in 40.2% of patients, with most being mild in severity. In conclusion, safinamide at a dosage of 100 mg/day significantly improved motor symptoms, QoL, and pain, and demonstrated a favourable safety profile without levodopa dosage escalation during the 18-week treatment period in Korean patients with PD.

*Trial registration number and date*: NCT05312632, First Posted: April 5, 2022

## Introduction

Parkinson’s disease (PD) is a progressive neurodegenerative disease associated with loss of dopaminergic neurons in substantia nigra and α-synuclein accumulation (Nogueira et al. 2024). The most common treatment for PD is replacement therapy using dopaminergic drugs, which includes levodopa and carbidopa (Gandhi et al. 2023). Levodopa is the most effective drug in most PD patients, improving motor symptoms related to the dopamine pathway (Ferreira et al. 2013; Fox et al. 2018). However, long-term use and high doses of levodopa can potentially lead to troublesome dyskinesia which can be difficult to treat (Kwon et al. 2022; Freitas et al. 2017). Also, as PD progresses, non-dopaminergic pathways (e.g., glutamate) become involved in the development of dyskinesia (Blandini et al. 1996). Therefore, there is a need for adjuvant therapy with both dopaminergic and non-dopaminergic effects during progression of PD (Borgohain et al. 2014).

Safinamide is a highly selective, reversible monoamine oxidase B (MAO-B) inhibitor that also reduces glutamate release (Müller 2018), and the phase 3 SETTLE study demonstrated the efficacy and safety of safinamide as add-on to levodopa in PD patients with motor fluctuations (Schapira et al. 2017). SETTLE demonstrated that safinamide has both dopaminergic and non-dopaminergic effect, resulting in significant improvements in motor symptoms and quality of life (QoL) in PD patients (Schapira et al. 2017). With respect to its dopaminergic effects, safinamide significantly improved resting tremor, addressing a limitation of levodopa, as levodopa has a less consistent effect on tremor than on bradykinesia and rigidity (Pirker et al. 2023). In terms of non-dopaminergic effects, the quality of life of PD patients, which is associated with numerous psychological and social problems as well as non-motor fluctuation (e.g., pain, mood), has also been improved by safinamide. However, the SETTLE study did not focus on the non-dopaminergic effects, particularly the glutamatergic pathway. Pain is a common and multifactorial condition in PD patients and has a significant negative impact on patients’ QoL (Nogueira et al. 2024).

We aimed to evaluate safinamide as add-on therapy, improving motor symptoms and patients’ QoL as well as pain. The multicentre, phase 4 KEEP (In South Korea, to Evaluate the Efficacy and safety of safinamide as add-on therapy to levodopa in Parkinson’s disease patients with motor fluctuation) study was designed to further evaluate the efficacy and safety of safinamide after 18 weeks as add-on therapy to levodopa in Korean PD patients with motor fluctuations. During the study, participants who could not escalate their levodopa dose, evaluated “off” time and “on” time without dyskinesia; non-motor symptoms such as pain and cognitive impairment; and QoL.

## Methods

### Study design

This was a prospective, multicentre, open-label, single-arm, interventional study, performed in South Korea, involving PD patients who were maintaining levodopa. The study commenced with a screening/wash-out period, during which patients who had previously taken medication such as catechol-O-methyl transferase (COMT) inhibitors and/or MAO-B inhibitors underwent an appropriate wash-out period for each medication (3 and 14 days, respectively; equivalent to more than five times the half-life of each medication).

During the treatment period, eligible patients received safinamide once daily for 18 weeks as an add-on therapy. All patients received a dose of 50 mg/day for the first 2 weeks, and 100 mg/day thereafter. Patients who were not able to tolerate 100 mg/day within 4 weeks were discontinued from the study. If adverse events (AEs) occurred after the dose was increased, it could be reduced back to 50 mg/day, and then increased back to 100 mg/day, both at the discretion of the investigator.

The levodopa dose was maintained at a constant level from screening. Reducing the dose was allowed, but there were no subjects who reduced levodopa dosage during the study period. Dopamine agonists being received at screening were also maintained at the same dose during the study period; if dopamine agonists were not being received at screening, they could not be initiated during the study period. Addition and dose adjustment of anticholinergic drugs and/or amantadine was at the discretion of the investigator. The use of acetylcholinesterase inhibitors, COMT inhibitors, deep brain stimulation, levodopa–carbidopa intestinal gel therapy, and surgical treatment were prohibited during the study.

Efficacy was assessed using PD diaries and validated questionnaire instruments at baseline and week 18. Safety was recorded at the same time.

The study was performed in line with the principles of the Declaration of Helsinki 2013, ICH Good Clinical Practice guidelines and Korean Good Clinical Practice guidelines. Approval was granted by the relevant Ethics Committees. Written informed consent was obtained from all study participants. The trial was registered at ClinicalTrials.gov (NCT05312632) on 5 April 2022.

### Study participants

Patients were required to meet the Movement Disorder Society (MDS) diagnostic criteria for Parkinson’s disease, have ≥ 1.5 h of “off” time daily, and to have received a stable dose of levodopa for ≥ 4 weeks prior to the screening. Patients had to take levodopa three or more times a day and maintain that dose during the 18-week treatment period without escalation. Moreover, dopamine agonists had to have been administered at a stable dose for ≥ 4 weeks prior to screening and be suitable for maintenance at that dose during the 18-week period without adjustment. In addition, patients had to have adequate cognitive function as determined by investigator’s judgement (or have a Global Deterioration Scale score ≤ 3 or a Clinical Dementia Rating of ≤ 0.5 within 3 months prior to screening) and be able to complete a patient diary.

The main exclusion criteria were previous history of medication such as COMT inhibitors and/or MAO-B inhibitors without wash-out (each wash-out period being 3 and 14 days, respectively); use of serotonergic medications or other medications for depression, or medications for psychosis, within 5 weeks prior to screening.

### Study assessments

Data on patient demographics and clinical characteristics, including medical history, prior and concomitant medication, height, weight, physical examination, urine pregnancy test (for females of childbearing potential), 12-lead electrocardiogram (ECG; if deemed necessary by the investigator), vital signs, laboratory tests (haematology, blood chemistry, urinalysis), and AEs, were collected during the screening and/or baseline assessment visits.

The co-primary endpoints were the change in daily “off” time from baseline to week 18 and the change in Parkinson’s Disease Questionnaire (PDQ-39) score from baseline to week 18. Patients completed PD diaries for 3 days prior to these visits to record “on” time, “on” time with dyskinesia, “off” time, and time asleep. The impact of PD on QoL (functioning and well-being) was assessed using the patient-completed PDQ-39, in which lower scores indicate better QoL (total score range, 0 ~ 156) (Jenkinson et al. 1997).

Secondary endpoints included: the change from baseline to week 18 in scores for the Movement Disorder Society-Sponsored Revision of the Unified Parkinson’s Disease Rating Scale (MDS-UPDRS) Part 3, MDS-UPDRS Part 4, King’s Parkinson’s Disease Pain Scale (KPPS) and Mini-Mental State Examination (MMSE); the change from baseline to week 18 in daily “on” time without dyskinesia; and safety. For Part 3, an observer scores the patient’s performance of specific physical tasks (total score range 0–132) (Goetz et al. 2008). Part 4 integrates clinical observations and patient-derived information to assess dyskinesia and motor fluctuations (total score range 0–24). Pain was assessed using the KPPS, with lower scores indicating less pain (total score range 0–168) (Chaudhuri et al. 2015). Cognition was assessed using the MMSE, with lower scores indicating worse cognition (total possible score 30) (Folstein et al. 1975).

Safety was assessed by evaluation of AEs throughout the study.

### Statistical methods

All efficacy analyses were performed using the Full Analysis Set (FAS) as the primary population. Safety assessments used the Safety Analysis Set (SAS). Data were summarized using descriptive statistics, including mean and standard deviation (SD) or standard error (SE) for continuous variables, and number and percentage for categorical variables. Changes from baseline in efficacy parameters were analysed using the paired *t *test for parametric data or Wilcoxon signed-rank test for non-parametric data. A significance level of 2.5% (two-sided test) was used for each of the co-primary efficacy endpoints (change in daily “off” time and change in PDQ-39 score). A significance level of 5% (two-sided test) was used for all secondary efficacy endpoints, including subgroup analyses.

The study sample size was calculated for both primary endpoints and the larger sample size (based on PDQ-39 score) was selected. Assuming efficacy would be similar to that observed in the phase 3 clinical trial, in which the average change in PDQ-39 score was − 3.17 (SD 10.86) (Schapira et al. 2017), and allowing for a 25% drop-out rate, a sample size of 199 was required to achieve 90% power with a significance level (alpha) of 0.025.

## Results

**Fig. 1:**
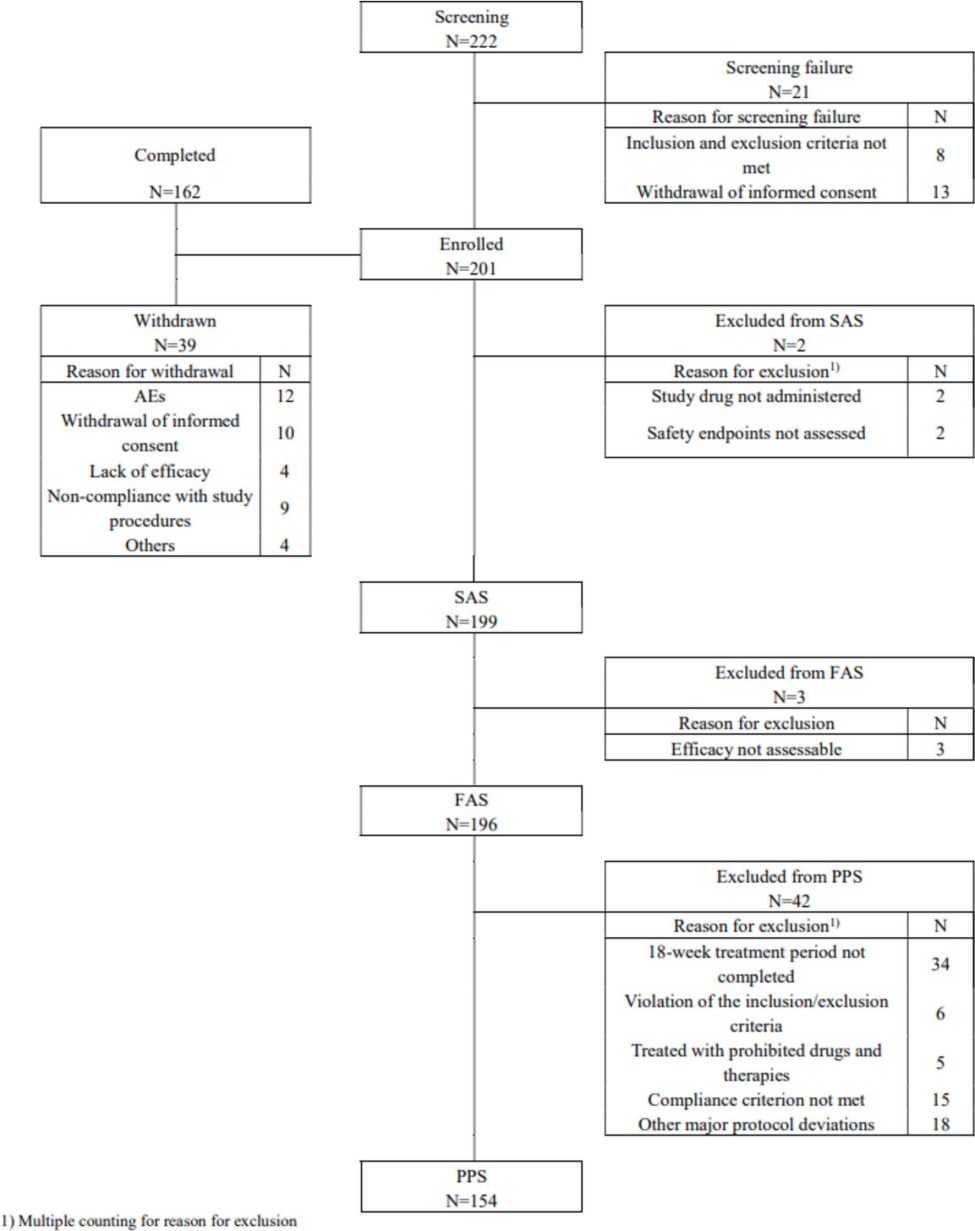
Disposition of patients

A total of 201 patients were enrolled from 20 centres between April 2022 and May 2023 (Fig. 1). Two patients were excluded from the safety analysis because study drug was not administered properly, and three were excluded from the efficacy analysis as relevant endpoints were not assessed.

At baseline, in the FAS (*n* = 196), 51.5% of the study population were female, the mean age was 63.7 ± 7.8 years (mean ± SD), the mean time since diagnosis of PD was 6.6 ± 3.7 years and the mean levodopa equivalent daily dose was 721.1 ± 297.4 mg (mean ± SD). The majority of patients (76.5%) were receiving stable doses of dopamine agonists (Table 1).

**Table 1:** Baseline characteristics (N = 196, full analysis set)

Age (years), mean ± SD	63.7 ± 7.8
Sex (male/female), n (%)	95 (48.5)/101 (51.5)
Duration of Parkinson’s disease (years), mean ± SD	6.6 ± 3.7
Mean daily dose of levodopa (mg), mean ± SD	502.4 ± 197.2
Mean daily dose of levodopa equivalent, mean ± SD	721.1 ± 297.4
PDQ-39 summary index, mean ± SD	24.9 ± 15.9
MDS-UPDRS Part 3 total score, mean ± SD	23.7 ± 13.3
MDS-UPDRS Part 4 total score, mean ± SD	6.0 ± 2.6
KPPS total score, mean ± SD	10.6 ± 14.4
MMSE total score, mean ± SD	27.8 ± 2.3
Concomitant medication, n (%)^a^	
Anti-Parkinson drugs	196 (100.0)
Dopamine agonists	150 (76.5)
Anti-cholinergic drugs	33 (16.8)
Amantadine	46 (23.5)

^a^Concomitant medications for managing Parkinson’s disease

During the study, treatment compliance was 95.3 ± 14.6%. Overall, compliance was in the range 80–120% for 180 (91.8%) patients, while compliance was < 80% in 15 (7.7%) patients and was > 120% in 1 (0.5%) patient.

### Efficacy

Despite not being allowed to escalate the levodopa dosage during the treatment period, at week 18 of safinamide add-on therapy, significant improvements from baseline were seen in the co-primary endpoints, mean daily “off” time (− 1.3 ± 2.4 h, *p *< 0.001) and QoL assessed by the PDQ-39 summary index (− 2.7 ± 10.3, *p *< 0.001) (Table 2). Among the PDQ-39 domains, significant improvements were seen in scores for the domains of Mobility (− 4.4 ± 18.7), Activities of Daily Living (− 5.4 ± 18.2), and Stigma (− 6.3 ± 23.2) (each *p *< 0.001).

**Table 2:** Change from baseline in daily “off” time and PDQ-39 score at week 18 (primary endpoints) (N = 196; full analysis set)

Parameter	Baseline	Week 18	Change from baseline	*P* ^(WS)^
Daily “off” time (h)	6.7 ± 2.9	5.4 ± 2.9^a^	− 1.3 ± 2.4	< 0.001*
PDQ-39 summary index	24.9 ± 15.9	22.3 ± 16.6	− 2.7 ± 10.3	< 0.001*
Mobility domain	30.5 ± 25.4	26.1 ± 25.3	− 4.4 ± 18.7	<0.001*
Activities of daily living domain	26.4 ± 23.3	21.1 ± 22.1	− 5.4 ± 18.2	< 0.001*
Emotional well-being domain	28.4 ± 24.5	29.5 ± 26.9	1.1 ± 21.0	0.932
Stigma domain	30.7 ± 27.9	24.4 ± 24.8	− 6.3 ± 23.2	< 0.001*
Social support domain	15.9 ± 16.0	14.5 ± 16.6	− 1.5 ± 13.9	0.131
Cognition domain	23.6 ± 21.0	21.7 ± 19.5	− 1.9 ± 17.1	0.322
Communication domain	17.3 ± 21.3	15.3 ± 21.0	− 1.9 ± 13.2	0.061
Bodily discomfort domain	26.6 ± 22.5	25.4 ± 21.7	− 1.1 ± 20.9	0.605

Data are presented as mean ± SD

*PDQ*-39 Parkinson’s disease questionnaire, *WS* wilcoxon signed rank test

*p value < 0.05

^a^ N = 189; 7 subjects were not assessed for their daily “off” time at week 18

At week 18, significant improvements were seen in motor symptoms assessed by MDS-UPDRS Part 3 (mean change in motor examination score − 1.7 ± 8.4, *p *< 0.001) and Part 4 (mean change in motor complications score − 0.7 ± 2.1, *p *< 0.001), and in daily “on” time without dyskinesia (1.2 ± 2.5 h, *p *< 0.001) (Table 3). At week 18, significant improvements were also observed in MDS-UPDRS Part 3 subgroups for bradykinesia, tremor and rigidity scores, and in MDS-UPDRS Part 4 subgroups for time spent in the off state, functional impact of fluctuations, complexities of motor fluctuations and painful off-state dystonia (Table 3). There was also a significant improvement in pain, based on the mean change in KPPS total score (− 1.5 ± 11.3, *p* = 0.013). Among KPPS domains, a significant improvement was seen only for Fluctuation-Related Pain (− 1.1 ± 5.4, *p* = 0.002). No significant improvement in cognition was seen, as assessed by the MMSE.

**Table 3:** Change from baseline in MDS-UPDRS Part 3 and part 4, KPPS and MMSE scores at week 18 (secondary endpoints) (N = 196; full analysis set)

Parameter	Baseline	Week 18	Change from baseline	*P* ^(WS)^
**MDS-UPDRS Part 3**^**a**^	23.7 ± 13.3	22.0 ± 13.9	− 1.7 ± 8.4	< 0.001*
Bradykinesia score (item 2, 4–8, 14)	12.2 ± 7.2	11.5 ± 7.6	− 0.7 ± 4.5	0.002*
Tremor score (item 15–18)	2.9 ± 3.6	2.5 ± 3.2	− 0.4 ± 2.7	0.024*
Rigidity score (item 3)	3.9 ± 3.3	3.4 ± 2.9	− 0.5 ± 2.4	0.002*
Axial symptom score (item 1, 9–13)	4.6 ± 3.4	4.5 ± 3.7	− 0.1 ± 2.6	0.309
Postural instability and gait disturbance score (item 9, 10, 12, 13)	3.3 ± 2.6	3.3 ± 2.8	− 0.1 ± 1.9	0.398
**MDS-UPDRS Part 4**^**b**^	6.0 ± 2.6	5.3 ± 2.8	− 0.7 ± 2.1	< 0.001*
Sum of dyskinesia-related scores (item, 1,2 = Part IVa)	0.9 ± 1.3	0.8 ± 1.4	− 0.0 ± 0.9	0.572
Time spent in the off state (item 3)	1.9 ± 0.8	1.6 ± 0.8	− 0.3 ± 0.7	< 0.001*
Functional impact of fluctuations (item 4)	1.7 ± 1.0	1.5 ± 1.0	− 0.1 ± 0.8	0.014*
Complexities of motor fluctuations (item 5)	1.2 ± 0.5	1.1 ± 0.5	− 0.1 ± 0.6	0.010*
Painful off-state dystonia (item 6 = Part IVc)	1.2 ± 0.5	0.3 ± 0.7	− 0.1 ± 0.6	0.004*
** KPPS, total score**	10.6 ± 14.4	9.1 ± 12.7	− 1.5 ± 11.3	0.013*
Musculo-skeletal pain domain	2.2 ± 2.7	2.5 ± 2.9	0.3 ± 3.0	0.432
Chronic pain domain	1.3 ± 2.9	1.0 ± 2.3	− 0.3 ± 2.7	0.126
Efficacy fluctuation-related pain domain	2.6 ± 6.0	1.5 ± 4.3	− 1.1 ± 5.4	0.002*
Nocturnal pain domain	2.0 ± 3.7	1.8 ± 3.9	− 0.1 ± 3.2	0.356
Oro-facial pain domain	0.4 ± 1.7	− 0.3 ± 1.4	− 0.1 ± 2.1	0.312
Discolouration, oedema/swelling domain	0.7 ± 2.6	0.5 ± 2.1	− 0.2 ± 2.5	0.394
Radicular pain domain	1.2 ± 2.4	1.3 ± 2.5	0.1 ± 2.5	0.516
**MMSE score**	27.8 ± 2.3	28.0 ± 2.3	0.2 ± 1.7	0.053
Daily “on” time without dyskinesia (h)	9.5 ± 3.1	10.7 ± 3.1	1.2 ± 2.5	< 0.001*

Data are presented as mean ± SD

*KPPS * King’s Parkinson’s disease pain scale, *MDS-UPDRS* movement disorder society-sponsored revision of the unified Parkinson's disease rating scale, *MMSE* mini-mental state examination, *WS* wilcoxon signed rank test

*p value < 0.05

^a^152 and 10 patients were assessed in the “on” and “off” states, respectively; the state was “not specified” for 34 patients

^b^151 and 11 patients were assessed in the “on” and “off” states, respectively; the state was “not specified” for 34 patients

### Safety

During the study, 80 patients in the SAS (40.2%, 80/199) experienced a total of 120 treatment-emergent AEs (TEAEs), with 50 patients (25.1%, 50/199) experiencing a total of 73 TEAEs that were considered related to the study drug. Most TEAEs were mild in severity (Table 4). The most common TEAEs were overdose (6.5%, 13/199), dyskinesia (5.5%, 11/199), COVID-19 (3.0%, 6/199), dizziness and nausea (each 2.5%, 5/199). Of the 11 patients with dyskinesia, none were taking amantadine. Fall was reported as an adverse event for one patient (0.5%, 1/199), and hypersexuality was reported for one patient (0.5%, 1/199). With respect to the AE ‘overdose’, all cases where treatment compliance exceeded 100% were classified as overdose and recorded as an AE. These cases generally appeared to be related to failure to properly carry out the dose adjustments specified in the protocol (initial dose escalation, or dose reduction for AEs).

**Table 4:** Treatment-emergent adverse events (TEAEs) (N = 199)

	n (%), [Number of events]	95% Confidence interval
Any TEAE	80 (40.2), [120]	(33.3–47.4)
Mild	78 (39.2), [115]	(32.4–46.4)
Moderate	5 (2.5), [5]	(0.8–5.8)
Severe	0 (0.0), [0]	(0.0–1.8)
Drug-related TEAEs	50 (25.1), [73]	(19.3–31.8)
Mild	50 (25.1), [73]	(19.3–31.8)
Moderate	0 (0.0), [0]	(0.0–1.8)
Severe	0 (0.00), [0]	(0.0–1.8)
Serious adverse events	4 (2.0), [5]	(0.6–5.1)
Serious drug-related adverse events	0 (0.0), [0]	(0.0–1.8)
Death due to TEAE	0 (0.0), [0]	(0.0–1.8)
Drug discontinuation due to TEAE	16 (8.0), [24]	(4.7–12.7)
TEAEs reported in ≥ 2% of patients		
Overdose^a^	13 (6.5) [13]	–
Dyskinesia	11 (5.5) [11]	–
COVID-19	6 (3.0) [6]	–
Dizziness	5 (2.5) [5]	–
Nausea^b^	5 (2.5) [5]	–
Drug ineffective	4 (2.0) [4]	–
Decreased appetite	4 (2.0) [4]	–

^a^All cases where treatment compliance exceeded 100% were classified as overdose and recorded as an adverse event.

^b^Vomiting (reported as a separate adverse event) occurred in 2 (1.0%) patients [2 events].

Four patients (2.0%, 4/199) experienced a total of five serious AEs (cartilage injury, muscle rupture, skin laceration, condition aggravated, COVID-19), none of which were considered drug-related. No deaths occurred. TEAEs led to discontinuation of the study drug in 16 patients (8.0%, 16/199), with the most common such events being ‘drug ineffective’ (2.0%, 4/199) and dyskinesia (1.0%, 2/199) of mild severity.

## Discussion

The KEEP study showed that treatment with safinamide added to levodopa reduced daily “off” time (i.e. time with decreased mobility, bradykinesia, or akinesia) and improved QoL in patients with PD with motor fluctuations in South Korea.

These findings add to the body of evidence from previous clinical trials demonstrating the efficacy and safety of safinamide in levodopa-treated PD patients with motor fluctuations (Borgohain et al. 2014; Schapira et al. 2017; Hattori et al. 2020; Wei et al. 2022), by providing additional data on safinamide add-on therapy specifically in Korean patients. Moreover, the aforementioned improvements with safinamide were demonstrated despite the relatively short treatment period and the inability to escalate the dosage of levodopa during the treatment period (18 weeks). Levodopa is the most effective drug to improve the motor symptoms of PD, however, high doses of levodopa promote levodopa-induced dyskinesia. A previous study demonstrated that higher cumulative levodopa dosage is associated with the earlier occurrence of motor complications including dyskinesia (Hauser et al. 2006). Hazard ratios that described the associations between subject characteristics and the time to first occurrence of dyskinesia were presented, and cumulative levodopa dose was significantly associated with earlier occurrence of dyskinesia (HR 1.19, 95% CI 1.08–1.31; *p *< 0.001) (Hauser et al. 2006). In that sense, the results of KEEP study are notable for showing a significant improvement in daily “on” time without dyskinesia even without an escalating levodopa dosage.

The efficacy data are consistent with those from previous studies. The mean decrease in daily “off” time at week 18 (co-primary endpoint) was 1.3 h. This is consistent with the decrease from baseline to 24 weeks of 1.56 h (mean difference versus placebo − 1.03 h, *p *< 0.001) seen with safinamide in the phase 3 SETTLE trial (Schapira et al. 2017) and 1.73 h in a study involving Japanese patients (mean difference versus placebo − 1.72, *p *< 0.0001) (Hattori et al. 2020). In studies with the second-generation MAO-B inhibitor, rasagiline (1 mg/day), the mean reduction in “off” time from baseline to 18 weeks was 1.18 h (difference versus placebo − 0.78, *p* = 0.0001) in LARGO (Rascol et al. 2005) and from baseline to 26 weeks was 1.85 h (difference versus placebo − 0.94, *p *< 0.001) in PRESTO (Parkinson Study Group 2005). In a phase 3 trial of the COMT inhibitor opicapone (50 mg/day), the mean decrease in “off” time after 15 weeks was 1.98 h (difference versus placebo − 0.91 h, *p* = 0.008) (Lees et al. 2017). The minimally important clinical difference in reducing time spent in the “off” state is about 1 h per day, as reported in a previous study (Rascol et al. 2019; Hauser et al. 2014). Therefore, our study demonstrated a clinically meaningful improvement in “off” time.

Noting that the minimally important difference (MID) of PDQ-39 (co-primary endpoint) was considered to be about 1.6 points based on previous studies (Peto et al. 2001), the reduction in PDQ-39 of 2.7 points in the current study confirmed a clinically meaningful improvement in patients’ QoL. Comparisons with other trials are limited by potential differences in patient profiles and study design; nonetheless, the change in PDQ-39 summary index in the current study appears consistent with the change from baseline to 24 weeks of − 3.17 (mean difference versus placebo − 2.33, *p* = 0.006) seen with safinamide in the phase 3 SETTLE trial (Schapira et al. 2017). Other studies have also found that safinamide add-on therapy was associated with improvements in QoL (Borgohain et al. 2014; Wei et al. 2022; Cattaneo et al. 2020). In a phase 3 study of the COMT inhibitor opicapone, the mean PDQ-39 score decreased by 4.4 points from baseline to 15 weeks, but this was not significantly different to the change of 4.8 seen with placebo (Lees et al. 2017). In the current study, reductions of 4–6 points in several PDQ-39 domains suggest that patients’ QoL improved most in terms of mobility, activities of daily living and stigma.

At week 18 in the current study, although not clinically significant (Schrag et al. 2006), statistically significant improvements from baseline were seen in motor symptoms assessed by MDS-UPDRS Part 3, including the subgroups for bradykinesia, tremor and rigidity scores. It is difficult to directly compare results because the assessment ranges between MDS-UPDRS part 3 and UPDRS part 3 differ. However, the efficacy seen with respect to motor symptoms is consistent with previous studies (Schapira et al. 2017, Lees et al. 2017).

The significant decrease in MDS-UPDRS Part 4 score in the current study, as well as significant improvements in the items of time spent in the “off” state, functional impact of fluctuations, complexities of motor fluctuations and painful off-state dystonia, indicates a reduction in motor complications with safinamide add-on therapy. In addition, the increase in daily “on” time without dyskinesia suggests that safinamide can improve patients’ physical function without causing movement disorders. The mean increase in daily “on” time without dyskinesia of 1.2 h after 18 weeks is consistent with the mean increase in “on” time without troublesome dyskinesia of 1.42 h (mean difference versus placebo 0.96, *p* < 0.001) after 24 weeks in SETTLE (Schapira et al. 2017), 1.66 h in a 24-week study in Japanese patients (Hattori et al. 2020), and 1.19 h in a 16-week study in Chinese patients (Wei et al. 2022). In the LARGO study of rasagiline, mean daily “on” time without troublesome dyskinesia increased by 0.85 h from baseline to week 18 (difference versus placebo 0.82 h, *p* = 0.0005) (Rascol et al. 2005). In the opicapone phase 3 study (50 mg/day), total “on” time increased by 1.86 h after 15 weeks (difference versus placebo 0.88 h, *p* = 0.005) (Lees et al. 2017).

Safinamide in combination with levodopa also improved non-motor symptoms in the current study, as indicated by an improvement in KPPS score, in particular fluctuation-related pain. This is consistent with data from previous studies that suggest safinamide has a positive effect on pain in PD patients with motor fluctuation (Cattaneo et al. 2017).

Cognitive impairment is common in patients with PD (Aarsland et al. 2021). In the current study, cognitive function did not change significantly during the study, based on MMSE scores in the FAS; however, the mean change in MMSE from baseline at 18 weeks was statistically significant in the PPS. The mean scores at baseline (27.8) and at week 18 (28.0) were above the cut-off score of 24 points that is considered to indicate there is no cognitive impairment (Trivedi et al. 2017). In SETTLE, mean MMSE score decreased slightly after 24 weeks, although the change was not significantly different to that seen in the placebo group (− 0.2 versus − 0.14, *p* = 0.26) and mean values at both baseline (28.66) and week 24 (28.46) were above the threshold of 24 (Schapira et al. 2017).

Previous clinical trials and observational studies have established that safinamide is generally well tolerated (Schapira et al. 2017; Borgohain et al. 2014; Hattori et al. 2020; Wei et al. 2022; Abbruzzese et al. 2021). Consistent with this, no significant safety concerns were identified in the current study, and most AEs were mild in severity. The incidence of AEs (40.2%) was lower than that in SETTLE (67.9%) (Schapira et al. 2017); however, the daily dose of levodopa was lower in the current study (502.4 mg/day) than in SETTLE (776.5 mg/day), which may account for some of the difference. Hypersexuality, which could result from a dopamine dose-dependent AE, was reported for only one patient (0.5%, 1/199) in the KEEP study. Dyskinesia is a possible AE with add-on therapy to levodopa, including COMT inhibitors and MAO-B inhibitors (Aradi and Hauser 2020). Dyskinesia is one of the most common AEs reported in studies of safinamide, with rates of 10–18% reported in phase 3 clinical trials (Schapira et al. 2017; Borgohain et al. 2014; Hattori et al. 2020; Wei et al. 2022), and a rate of 13.7% reported for a large observational study in routine practice in which most patients (92.2%) had motor fluctuations (Abbruzzese et al. 2021). The incidence of dyskinesia in the current study was lower, at 5.5% (11/199), and all events were mild; none of the patients reporting dyskinesia were receiving amantadine. It is possible that this difference could, in part, be explained by a lower mean daily dose of levodopa in the current study; for example, as noted earlier, the mean daily dose of levodopa in SETTLE was higher than in the current study, as was the incidence of dyskinesia (14.6%) (Schapira et al. 2017). The incidence of dyskinesia in phase 3 studies of rasagiline 1 mg/day was 5–18% (Parkinson Study Group 2005; Rascol et al. 2005) and with opicapone 50 mg/day was 24.0% (Lees et al. 2017). In other words, the KEEP study demonstrated the efficacy of safinamide without exacerbating dyskinesia. Additionally, safinamide could be used for patients experiencing dopamine dose-dependent AEs, such as hypersexuality and alcohol abuse disorder, by maintaining their levodopa dosage (De Micco et al. 2022).

The main limitation of the study is the open-label, single-arm design, with the lack of a control group precluding precise evaluation of the efficacy and safety of the drug (including any potential reward expectation effect). However, it does provide supportive evidence of the efficacy and safety of safinamide added to levodopa in PD patients with motor fluctuations. Although indirect comparison of study results should be interpreted with caution, the results obtained in Korean patients are consistent with those from the global phase 3 trial of safinamide (Schapira et al. 2017), as well as studies in Japanese (Hattori et al. 2020) and Chinese patients (Wei et al. 2022). Additionally, this study aimed to evaluate the efficacy and safety of safinamide when maintained at 100 mg/day after a 2-week administration at 50 mg/day; however, dose reduction for AEs was allowed, and 20 subjects reduced their dosage to 50 mg/day during the treatment period, with only one among them escalated back to 100 mg/day. As a result, there was a limitation in fully assessing the efficacy and safety of safinamide at 100 mg/day. In some cases, at the discretion of the investigator, patients were administered anti-cholinergics (16.8%) or amantadine (23.5%) to potentially alleviate symptoms of muscle rigidity and dyskinesia, and this may have confounded the results. In particular, the potentially dyskinesia-abating effect of amantadine may have confounded the dyskinesia findings in the 46 patients who received amantadine in the current study. Furthermore, although previous studies have reported that safinamide improved sleep and daytime sleepiness (Santos García et al. 2022) but failed to provide evidence of improved apathy (Kulisevsky et al. 2022) in PD patients, these parameters were not assessed in the current study. A strength of the study is that it provides data specifically for Korean patients, which is important to understand the generalizability of the effect of safinamide across different PD populations, as only a small number of patients from South Korea were included in the global phase 3 trials. The study used validated instruments to assess efficacy. In addition, it required patients to remain on stable doses of levodopa (reduction was allowed, but no cases were observed) and dopamine agonists throughout the study, to avoid a confounding effect.

## Conclusion

Safinamide, at the dosage of 100 mg/day, significantly improved motor symptoms, QoL, and pain in Korean PD patients with motor fluctuations, and was generally well tolerated, without levodopa dosage escalation during the 18-week treatment period.

## Data Availability

The data that support the findings of this study are not openly available due to reasons of sensitivity and are available from the corresponding author upon reasonable request.
